# Midgut Volvulus on an Octogenarian Male: A Case Report

**DOI:** 10.7759/cureus.41667

**Published:** 2023-07-10

**Authors:** Enrique J Ramirez-Merced, Gabriela E Arizmendi-Velez, Rishi Sharma, Jesus E Guarecuco Castillo, Rana El-Tawil, Mohammad M Masri

**Affiliations:** 1 General Surgery, University of Medicine and Health Sciences, Camps, KNA; 2 General Surgery, D.Y. Patil Medical College, D.Y. Patil Education Society (Deemed to be University), Kolhapur, IND; 3 General Surgery, Larkin Community Hospital, South Miami, USA

**Keywords:** internal herniation, midgut malrotation, elderly patients, surgical gastroenterology, surgical case report, lower gi surgery, volvulus of midgut

## Abstract

Midgut volvulus is a rare incidence in adults, especially in octogenarians. More unlikely is to find a midgut volvulus without necrosis or the need to do bowel resection when the volvulus is found within an internal hernia due to a mesenteric defect. No case has been reported with our unusual presentation, making it a rare and challenging discovery. We describe the case of an 83-year-old male who presented with nonspecific symptoms and was found to have a midgut volvulus with an internal hernia through a mesenteric defect, which had a successful recovery at the end.

## Introduction

Midgut volvulus is a torsion of the small bowel that, upon diagnosis, is a surgical emergency due to vascular compromise of the abdominal area. Midgut volvulus is mostly found in infants due to congenital gut malrotation during embryogenesis [1]. Because of this, midgut volvulus is more commonly found in children and a rare finding in adults. It could present as a vague abdominal complaint in adults, or they can be asymptomatic. Incidence is estimated at 0.2% in adults [2]. This case came a little more unusual as an internal herniation of the small bowel through the mesenteric defect was found during the surgery. Volvulus is a condition in which a loop of the intestine has twisted 180° or more on its own, leading to obstruction of the loop. This is often accompanied by a compromise of the vascular supply to the loop. Small bowel volvulus may be caused by a primary (rare) condition or secondary to various other factors (common). Predisposed conditions may include malformation, congenital bands, postoperative adhesive adhesions, tumors, intussusceptions, or internal hernias. Diagnosing the condition as soon as possible is essential, as mortality rates can be as high as 67% with delayed treatment. Mortality and morbidity are caused by vascular compromise with ischemia and necrosis of the bowel [3]. 

In this case report, we discuss an elderly male patient with an unusual midgut volvulus at the age of 83 years who presented with nonspecific symptoms, and imaging results showed small bowel volatilization around the superior mesenteric artery (SMA) due to internal herniation secondary to a mesenteric defect, which, with a lot of difficulties, we managed to retrieve the patient's baseline functioning within three weeks postoperatively. Thus, a high index of suspicion and a prompt surgical exploration for its correction are essential.

## Case presentation

The patient was an 83-year-old Hispanic male with a past medical history of hypertensive disorder, pulmonary fibrosis, chronic obstructive pulmonary disease (COPD), gastroesophageal reflux disease (GERD), and psychotic disorder. Past surgical history consisted of bilateral hip arthroplasties. The patient presented to the Emergency Department from an Assisted Living Facility (ALF) due to respiratory distress. The patient was a poor historian and denied any symptoms or findings. He was given solumedrol IV (corticosteroid) and bronchodilator therapy, where his respiratory distress improved. Upon evaluation by the internal medicine service, the patient was admitted for further observation. 

**Figure 1:**
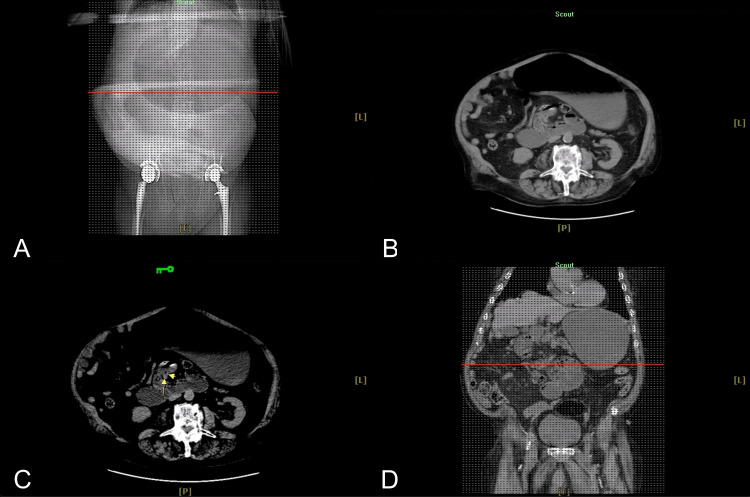
(A)-(D) Abdominopelvic CT scan without contrast shows midgut volvulus. Impressions: Fluid-filled distal esophagus and severely distended stomach with an air-fluid level. There is distended duodenum and proximal portion of the jejunum with the collapse of the rest of the small bowel and twisting of the superior mesenteric vessels. Small fat-containing ventral abdominal wall hernia in the suprapubic region. CT, computed tomography

**Figure 2:**
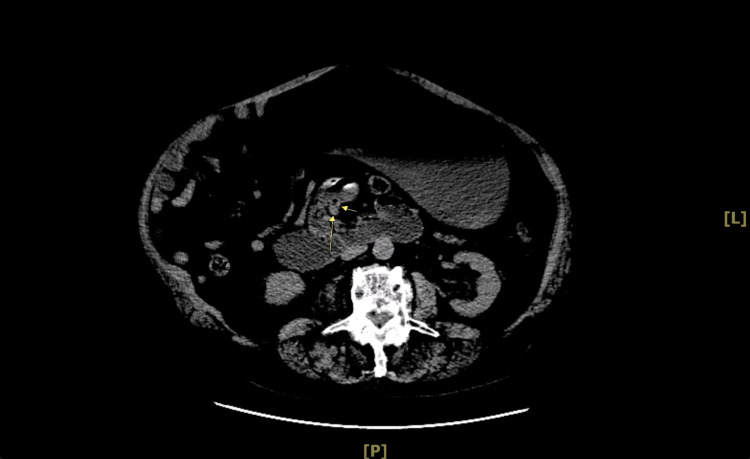
Abdominopelvic CT scan without contrast results. Yellow arrows: The whirl-like appearance of the bowel around the SMA. SMA, superior mesenteric artery; CT, computed tomography

During his progression in observation, the patient was found to have gradual distension of his abdomen as well as associated abdominal discomfort. The primary team initiated the placement of a nasogastric tube (NGT) and computed tomography (CT) scan to rule out any intra-abdominal pathology. General surgery was consulted immediately after the findings of a significantly severely distended stomach as well as a fluid-filled distal esophagus with a distended duodenum in the proximal portion of the jejunum with the collapse of the remainder of a small bowel and concern for twisting of the superior mesenteric vessel and the mesentery showing some fat stranding and concern of midgut volvulus (Figures 1-2).

Upon evaluation by the surgical team, the patient was found with abdominal distension and discomfort. Surgical intervention was discussed for the need for an exploratory laparotomy. The proxy signed informed consent.

**Figure 3:**
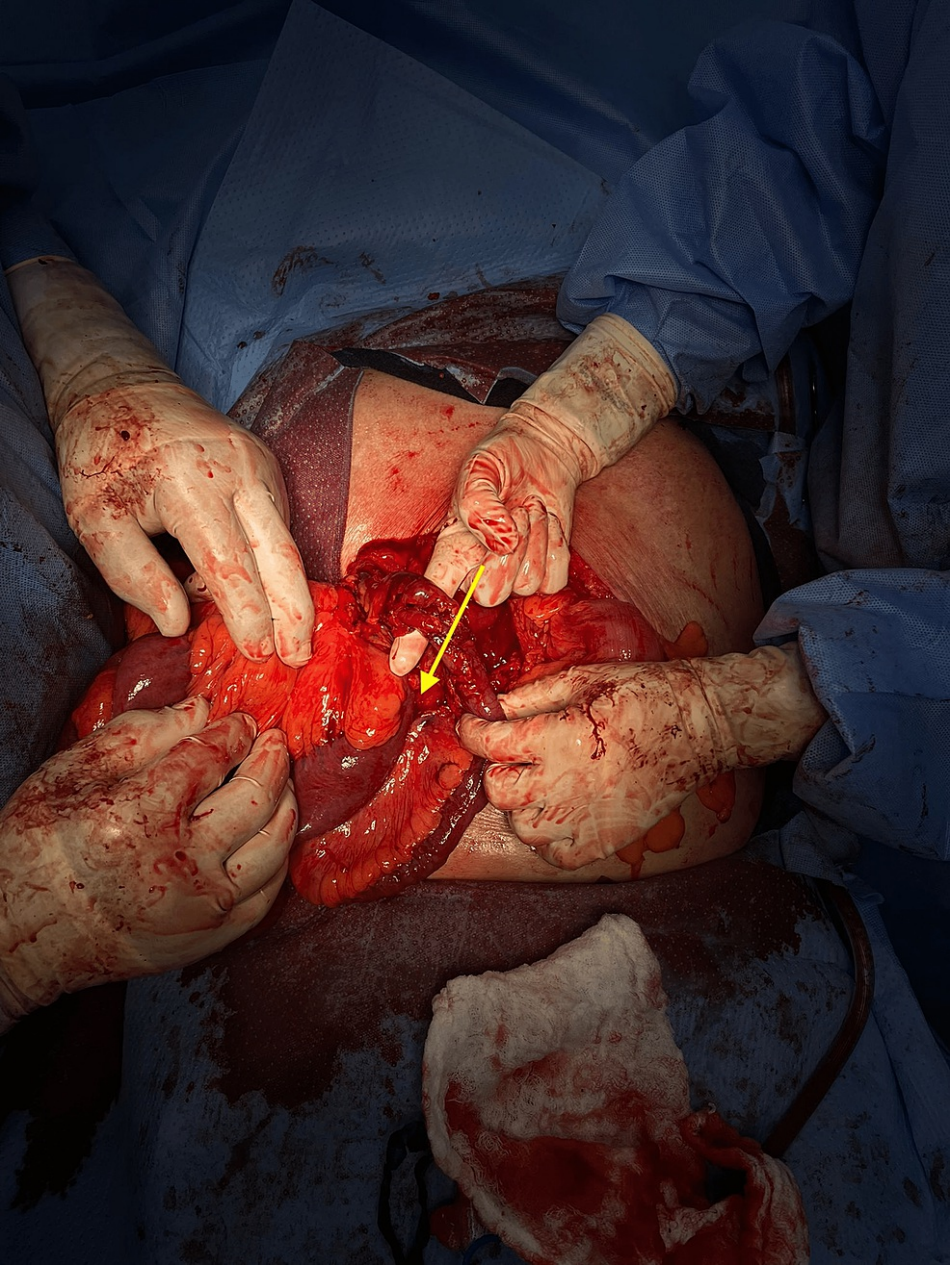
Internal herniation of the small bowel through the mesenteric defect. Yellow arrow: Herniation of the small bowel trajectory.

We initiated our procedure by performing a midline laparotomy incision. We initially noted a significant decompressed small bowel. We initiated proximally at the ligament of Treitz to run the bowel upon our meticulous approach for the running of the small bowel of the jejunum, where we noticed that a large internal herniation of the majority of the small bowel went through a mesenteric defect. The terminal ileum was surrounded by dense adhesions at the root of the mesentery of the small bowel, in the area of herniation. Additionally, the small bowel was rotated greater than 180° around the engorged superior mesenteric pedicle (Figure 3). We also found multiple areas of edema and inflammation in the mesentery, which perpetrated the internal herniation and the volvulization of the small bowel.

There were adhesions at the root of the small bowel, tightly encasing and involving the adjacent small bowel mesentery. By meticulous dissection, we freed the terminal ileum and the ileal segment from the surrounding mesenteric adhesions, and a defect was then clearly visualized. The reduction of the internal herniation and volvulization of the small bowel was performed carefully until the entirety of the bowel was devolvulized. The mesenteric defect was then approximated using 3-0 Vicryl stitches in a continuous running suture. We reexamined and ran the small bowel to ensure appropriate anatomical placement.

We began the procedure at the level of the ligament of Treitz, taking a meticulous approach to trace the small bowel down to the terminal ileum. During this examination, we ensured that no additional mesenteric defects were identified. However, further examination of the small bowel raised concerns regarding its perfusion. As a result, a decision was made to perform a small bowel resection of approximately 10 cm using a linear GIA stapler. Subsequently, an end-to-end stapled anastomosis was created. The patient had no perioperative complications. The patient remained intubated and was taken to the intensive care unit (ICU) for postoperative care. 

Six days after the procedure was done, a small bowel series demonstrated normal course and transit contrast material through the small and large bowel loops with no evidence of small bowel obstructions. During the patient's stay at the ICU, several attempts were made to start the patient on oral feedings. The patient failed his first barium swallow test. After 48 hours, the test was repeated, and this time he successfully passed. After 20 days, the patient was transferred to the medicine floor, where the trickle feedings started, and he was later discharged to a nursing home for long-term care.

## Discussion

Midgut volvulus is more commonly presented in infants and children but rarely in adults. It is roughly estimated that the incidence of this disease in adults is 0.2% to 0.5%, which decreases with age. Clinical presentations of midgut volvulus in adults are rare. Some adults may be asymptomatic, which may lower the incidence rate of the disease. It has been documented in previous cases that the disease can present with recurrent pain, intermittent obstruction, and malabsorption over months to years [2,4]. These symptoms in adults can be variable, and preoperative diagnosis of midgut volvulus may be challenging. Acute midgut volvulus is a surgical emergency where time defines mortality due to possible complications such as vascular compromise causing bowel ischemia and necrosis of the bowel, obstruction, and even death [2]. Mortality rates have been demonstrated to be as high as 67% with delayed treatment of the disease [3]. These incidence rates may increase over time due to advanced imaging, where currently, a CT scan is the first line of choice when it comes to imaging because it would show the *whirl sign*, which is a hallmark of midgut volvulus. The *whirl sign* denotes the pattern of the mesentery, and SMV is rotated clockwise around the SMA [4,5]. Cases have been reported in adult life, but the clinical presentation with the age of our patient was entirely unusual.

A small bowel volvulus can be primary (rare) or secondary to many other factors (usual). Predisposing conditions include malrotation (failure of the bowel to undergo counterclockwise rotation during embryogenesis), congenital bands, postoperative adhesions, tumors, intussusception, or internal hernia [3,6]. Shetty and Nayak stated that certain positional congenital anomalies can cause the jejunum and ileum to be in the upper left part of the abdominal cavity, where this congenital anomaly can cause volvulus of the colon at any age [7]. We did not find any previous history of abdominal wall malformation in this patient. Still, in the operating room, we found he had a mesenteric defect on which an internal hernia was formed. The findings we encountered in this patient, midgut volvulus and internal hernia due to a mesenteric defect, were a medical emergency that has not been presented in the literature. Based on this, this presentation of midgut volvulus would be classified as a secondary midgut volvulus by findings of internal hernia due to the mesenteric defect. The rarity of this case presented a challenge for our team, as the state of the volvulus and the patient's age were not promising for a successful operative intervention. The annual incidence rate of this condition is reported to be 1.7-5.7 per 100,000 of the population, with rare occurrences in the Western world. However, it is more commonly encountered in the Eastern world [8]. In the Eastern world, this condition is more commonly observed in regions with lower socioeconomic status. It is often associated with the practice of consuming large, indigestible meals after periods of fasting, as well as mesentery hypermobility [9].

Some physical examination findings of a patient with advanced midgut volvulus are concerning and suggest major complications. Coste et al. stated that signs of peritonitis may be suggestive of intestinal ischemia with edema and erythema of the abdominal wall. The most prevalent symptom in older children and adults is the abrupt onset of abdominal pain. The abdominal pain can last hours to days or can be chronic intermittent pain for weeks, months, or years. Other symptoms include chronic diarrhea, intermittent vomiting, malabsorption, and failure to thrive. If the patient is found to have a necrotic bowel, it can be conservatively resected to ensure adequate length for feeding and preventing short gut syndrome. Delaying the diagnosis of midgut volvulus can lead to serious consequences such as short gut syndrome requiring intestinal transplantation or death [1]. 

A surgical Ladd's procedure is the surgical intervention used in cases like this. This procedure consists of four parts: detorsion of any midgut volvulus in the counterclockwise direction; division of Ladd's bands overlying the duodenum, relieving the source of intermittent obstruction; mobilization of the duodenum to widen the narrowed small bowel mesentery; and division of adhesions around the SMA to prevent further volvulus. In cases where there is malfixation and the small bowel is normally located, currently, there are no identified surgical means to prevent midgut volvulus. There is a high risk of these patients developing complications, such as bowel obstruction, in the future [5]. According to Haak et al., performing Ladd's procedure or ordinary Ladd's bands along with adhesiolysis has been shown to reduce symptoms and prevent the further progression of the disease, most likely due to the new abdominal adhesions [5,10].

## Conclusions

To date, we could not find a case presenting a mesenteric defect with internal herniation and small bowel volvulization around the SMA without signs of necrosis. This is the first reported case of its kind, involving an octogenarian male who presented to the emergency department with nonspecific symptoms and survived the extent of the surgical procedure without complications. A possible cause could have been the mesenteric defect found in the operating room, where there were findings of internal herniation. This case shows the importance of advanced imaging technology such as CT scans for proper diagnosis and treatment. The procedure of choice for midgut volvulus will be Ladd's procedure, where the procedure reduces symptoms and helps prevent further development of this disease. Furthermore, the importance of documenting these types of cases is to show that they do occur in adult patients and it is important to diagnose and treat them appropriately. We aim to ensure that physicians recognize this rare presentation and decrease the morbidity and mortality in adults with midgut volvulus.
